# Recent Development of Rapid Antimicrobial Susceptibility Testing Methods through Metabolic Profiling of Bacteria

**DOI:** 10.3390/antibiotics10030311

**Published:** 2021-03-17

**Authors:** Chen Chen, Weili Hong

**Affiliations:** Institute of Medical Photonics, Beijing Advanced Innovation Center for Biomedical Engineering, School of Biological Science and Medical Engineering, Beihang University, Beijing 100191, China; chenxiaocuoao@buaa.edu.cn

**Keywords:** AST, metabolism, bacteria, antibiotic, resistance

## Abstract

Due to the inappropriate use and overuse of antibiotics, the emergence and spread of antibiotic-resistant bacteria are increasing and have become a major threat to human health. A key factor in the treatment of bacterial infections and slowing down the emergence of antibiotic resistance is to perform antimicrobial susceptibility testing (AST) of infecting bacteria rapidly to prescribe appropriate drugs and reduce the use of broad-spectrum antibiotics. Current phenotypic AST methods based on the detection of bacterial growth are generally reliable but are too slow. There is an urgent need for new methods that can perform AST rapidly. Bacterial metabolism is a fast process, as bacterial cells double about every 20 to 30 min for fast-growing species. Moreover, bacterial metabolism has shown to be related to drug resistance, so a comparison of differences in microbial metabolic processes in the presence or absence of antimicrobials provides an alternative approach to traditional culture for faster AST. In this review, we summarize recent developments in rapid AST methods through metabolic profiling of bacteria under antibiotic treatment.

## 1. Introduction

The emergence of antibiotic resistance is rising and has become a global public threat [1,2,3]. This crisis is aggravated by the overuse and misuse of antibiotics, especially the unnecessary use of broad-spectrum antibiotics [4,5,6,7]. To decrease the need for broad-spectrum antibiotics and treat an infection with appropriate drugs, the antimicrobial susceptibility of infecting bacteria needs to be determined in the early stage of infection to make a personal treatment plan [8]. Meanwhile, early treatment has relevance to the decrease of the death rate [9,10,11,12]. Therefore, rapid antimicrobial susceptibility testing (AST) is essential for treating infections with appropriate antibiotics, which will reduce the death caused by antibiotic-resistant bacteria and slow down the emergence of antibiotic resistance consequently.

Traditional AST methods, including broth microdilution, agar dilution, and disk diffusion methods, are based on the differential growth of bacteria in the presence or absence of antimicrobials. These methods are generally reliable and have been highly standardized and recommended by the Clinical and Laboratory Standards Institute (CLSI) and the European Committee on Antimicrobial Susceptibility Testing (EUCAST) as gold-standard methods. However, these methods are often slow, requiring at least a whole day to get a reliable readout and an additional 1−2 days for clinical samples [13]. Commercial automated AST systems, such as VITEK bioMérieux, MicroScan WalkAway, BD phoenix, and Sensititre, avoid complex steps in conventional methods and reduce the time to 4.5−24 h [14]. However, these systems still require bacterial isolates derived from an overnight culture, and thus the results cannot be obtained until the next day for clinical samples [13]. There is an urgent need for new technologies that can perform AST rapidly.

Microbial metabolism has shown to be related to antibiotic resistance [15]. For example, metabolite alterations affect stress adaptability and antibiotic resistance of microorganisms [16,17]. Furthermore, the metabolic cycle of bacteria is much shorter than the time of multigeneration culture. Therefore, detecting microorganisms’ metabolic response to drugs provides a new idea to shorten the turnaround time of AST. There are many reviews focusing on different aspects of rapid AST development with different techniques [14,18,19,20,21,22]. In this article, we summarize and review the recent development of rapid AST methods based on the bacterial metabolic response to antibiotic treatment.

## 2. Rapid AST Methods through Metabolic Profiling of Bacteria

### 2.1. Adenosine Triphosphate (ATP) Bioluminescence-Based Methods

ATP is the most common compound representing energy transfer in cell metabolism. Its level has a strong positive correlation with the number of bacterial cells [23]. As a result, measuring the ATP level as a reflection of bacterial growth would be a more quantitative method than the turbidity method with optical density measurement used in the conventional broth microdilution method. ATP bioluminescence, produced by a biochemical reaction that utilizes ATP in the oxidation of luciferin to adenyloxyluciferin and releases photons, can measure the ATP level with a luminometer. Many studies on using this technology for rapid AST have been demonstrated and can obtain AST results in 2 to 4 h [24,25,26,27].

McWalter et al. reported a rapid method for determining the susceptibility of *Staphylococcus aureus* through the differences in ATP values between control and methicillin-treated groups [24]. They found an incubation time of 2.5 h is optimal for determining the susceptibility. For methicillin-resistant strains, there was no significant decrease in ATP values. In contrast, ATP values showed a sharp drop for the susceptible strains. The susceptibility results obtained on testing 50 strains of *S. aureus* correlate precisely with the disk diffusion and the MIC results obtained by the agar dilution method [24]. Later, a rapid AST method based on AST luminescence was demonstrated in other bacterial species, including *Enterobacteriaceae* and Gram-positive cocci. The AST results (susceptible or resistant) by the ATP luminescence assay took about 4 h and correlated well with the overnight MIC values for most bacteria tested. However, a significant number of false results (13% *S. aureus* strains) were observed for *S. aureus* tested with penicillin by the ATP method [25,26].

Aside from cell lysis induced by antibiotics, bacteria also release ATP into the extracellular matrix during bacterial growth. It was reported that bacteria release ATP into the extracellular matrix during the log phase of bacterial growth, then the ATP released decreases after the stationary phase. This increasing and decreasing ATP/OD_600_ value was detected in many types of Gram-positive and Gram-negative bacteria [28]. Therefore, distinguishing between ATP release due to cell growth and ATP release due to cell lysis using this trend can better perform quantitative susceptibility testing. Heller et al. developed a rapid AST that utilizes the determination of ATP release from bacteria and the overall OD_600_ measurement. In this work, antibiotics (levofloxacin, gentamicin, kanamycin) were added to the bacterial solution when the bacteria were in the growth stage after 2-h incubation [29]. If an antibiotic was effective, bacteria were lysed in the presence of the antibiotic, resulting in an increase in the release of intracellular ATP from bacteria to the extracellular matrix. For susceptible strains, a significant increase in the ATP/OD_600_ ratio between the antibiotic-treated and the control groups was detected within 40 min for *Bacillus subtilis* and 20 min for *Escherichia coli*. For resistant strains, no increase in the ATP/OD_600_ ratio was detected. Compared to the approach mentioned above, this assay took a shorter antibiotic incubation time; however, this approach includes a 2-h pre-cultivation before the addition of antibiotics, resulting in a total assay time of around 3 h. Moreover, bacterial samples used in these studies were all isolated and purified strains since the quantification of bacterial ATP was interfered with by the non-bacterial ATP present in clinical samples.

Ivancic et al. reported the first use of the ATP bioluminescence method to perform AST of bacterial pathogens in clinical samples [27]. In this study, ATPase was used to eliminate human sources of ATP in clinical urine samples. Then, a bacterial-specific ATP bioluminescence assay was performed to estimate bacterial density in the samples. After a 2-h incubation, the ATP bioluminescence assay was performed again to evaluate bacterial response to antibiotic treatment. By quantification of bacterial bioluminescence with or without antibiotic treatment, the susceptibility of bacteria could be obtained within 2 h, with an overall accuracy rate of 91% for a total of 104 clinical urine samples. This method has a relatively high sensitivity compatible with clinically accepted cutoffs for urinary tract infections (UTI, 10^5^ colony-forming units mL^−1^) and therefore can apply directly to clinical urine samples. However, this method’s accuracy depends on the growth of bacteria; if the increase in bacterial ATP change in the antibiotic-free growth medium is less than 10-fold, the accuracy of antibiotic susceptibility prediction is only 43%. Moreover, this method could only determine whether the bacteria were resistant or susceptible; it did not provide MIC values.

Compared to urine specimens, blood culture specimens contain blood corpuscles and therefore present a big challenge for direct AST. The abundant intracellular ATP level derived from blood corpuscles could be a big obstacle for detecting the ATP level in bacteria. To overcome this challenge, Matsui et al. adjusted the test procedure to eliminate the impact of ATP released by blood cells and developed a rapid method based on ATP bioluminescence to perform AST directly from positive blood cultures [30]. The background ATP was significantly reduced by centrifugation to remove the blood corpuscles and followed by the addition of ATP-eliminating reagent to the supernatant bacterial suspension. This adjusted procedure reduced the background ATP by more than five orders of magnitude. With this method, ATP measurement was possible for positive blood culture specimens after a simple 15-min procedure. In addition, the MIC value could be determined after 6 h of incubation with this approach. However, overall, the obtained MIC values tended to be higher than those of the conventional broth microdilution method. In a total of 15 clinical specimens tested against levofloxacin, the MIC values of 5 specimens were higher than those of the conventional method. More tests need to be performed to validate the accuracy of this approach.

The advantages of ATP bioluminescence-based approaches are the simplicity of the methods and the low cost of the instruments. Although these methods need an ATP measurement device and luminescence reagent, they do not require experienced staff to perform the test. Therefore, these assays have the potential to be used in the clinic for rapid AST, but more tests on different species and antibiotics need to be performed to validate and refine these methods.

### 2.2. Nucleic Acid-Based Biochemical Methods

Nucleic acid-based technology is an important molecular method in the field of microbial research. For example, genotypic AST methods based on the detection of known resistance genes are highly sensitive and do not depend on bacterial culturing [31,32,33,34]. However, genotypic methods only detect specific known genetic sequences with resistance; they could not detect resistance with new mechanisms. Furthermore, the presence of certain resistance genes or mutations does not necessarily translate into phenotypic resistance [35]. Therefore, it is not a universal solution to predict antibiotic resistance by analyzing some known resistance genes [36,37]. To overcome these limitations, the development of phenotypic AST methods that quantitatively measure nucleic acid changes after drug exposure provides a potential solution [38,39,40,41].

For example, quantitative detection of rRNA has been used for AST. rRNA is an excellent target for pathogen detection because of its abundance in bacterial cells. As antibiotics affect the metabolism of antibiotic-susceptible bacteria from the log phase to the stationary phase, the cellular precursor rRNA level is expected to decrease [42]. Halford et al. developed a rapid AST platform by detecting and quantifying precursor rRNA using electrochemical sensors [43]. Precursor rRNA, an intermediate stage in the formation of mature rRNA, was used as a marker for cellular metabolism and growth rate. The results proved that for antibiotics that affect DNA and RNA syntheses, exposure time as short as 15 min would affect *E. coli* transcription [43]. However, antibiotics tested in this work differ in their effects on pre-rRNA and mature rRNA. For example, while the addition of rifampin caused a selective drop in pre-rRNA, chloramphenicol’s addition caused a selective increase in pre-rRNA; in contrast, the addition of gentamicin did not affect the level of pre-rRNA. Therefore, antimicrobial susceptibility may need to be determined case by case. Different calculation approaches are necessary to obtain the AST results. Thus, due to the different mechanisms of antibiotics, this method needs more tests.

Real-time polymerase chain reaction (PCR) has been developed for rapid AST by monitoring the amount and copy number of the highly conserved 16S rDNA gene of bacteria after antibiotic treatment [39]. Lee et al. described a method that determines susceptibility by applying real-time PCR to monitor bacterial load with the highly conserved 16S rRNA gene [44]. This method works for blood samples, as demonstrated for *E. coli* spiked in blood samples. Susceptibility was determined by monitoring the cycle threshold difference in bacterial load between treated and untreated samples. Three strains of *E. coli* were tested toward spectinomycin, chloramphenicol, and kanamycin in this study. Furthermore, MIC was successfully determined for blood samples spiked with *E. coli* susceptible to spectinomycin. With this method, antimicrobial susceptibility, MIC, and pathogen identification can be obtained for bacteria in blood in less than 24 h.

**Figure 1:**
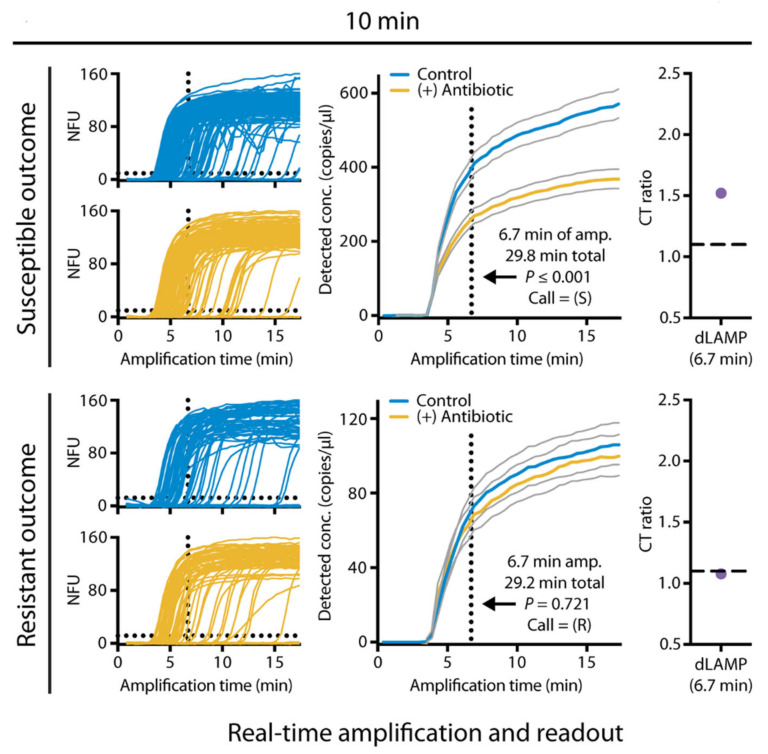
Sample-to-answer antimicrobial susceptibility testing (AST) in less than 30 min for *Escherichia coli* in urine using digital loop-mediated isothermal amplification (dLAMP). Data for one resistant and one susceptible sample are shown. dLAMP was monitored in real-time, and a susceptibility call was determined after 6.7 min of amplification. Gray lines represent 95% confidence intervals. Reprinted from reference [31] with permission.

However, the detection time of more than ten hours using PCR is still long for clinical application. To develop more rapid test methods, digital PCR was used to shorten the measurement time. Schoepp et al. reported a rapid phenotypic AST method by measuring DNA concentration using digital PCR [45]. In this work, digital PCR was used to divide bacterial chromosomal DNA into thousands of compartments. Then, it used targeted amplification to determine the number of "positive" compartments that contain the target genes. Compared to conventional PCR, digital PCR is more accurate and faster in measuring bacterial DNA concentration [46,47]. This method shortens the antibiotic exposure time to 15 min and reduces the measurement time to 2 h using a commercial droplet digital PCR [45]. In subsequent work by the same group, this method was used to perform rapid AST directly from clinical urine samples [31]. In this work, a rapid digital loop-mediated isothermal amplification (dLAMP) assay was developed and further reduced the measurement step to less than 10 min (Figure 1). To perform AST directly from clinical samples, a clinical urine sample was divided into two equal volume samples, one with antibiotic and control without antibiotic, and incubated for 15 min. The dLAMP then quantified the AST marker of a target nucleic acid sequence after incubation. The susceptibility (susceptible or resistant) was determined by the ratio of the marker concentrations in the control and antibiotic-treated samples with a susceptibility threshold. This approach achieved a categorical agreement of 98.1% for a total of 54 tests in clinical *E. coli*-infected UTI samples. With the ultrafast measurement, the sample-to-answer time of AST is possible in less than 30 min for *E. coli* [31].

The advantage of nucleic acid-based assays is the rapidity, especially for digital PCR. Moreover, universal phenotypic AST is possible by quantification of the copy number of DNA. However, nucleic acid-based assays require pre-knowledge of the resistance alleles, which is often poorly known. Furthermore, most of these assays demonstrated the resistant or susceptible classification only without the exact MIC value. Although the digital PCR devices are expensive and require experienced personnel for the operation, the application of devices based on isothermal amplification may change the situation and reduce the cost.

### 2.3. Matrix-Assisted Laser Desorption Ionization Time-of-Flight Mass Spectrometry (MALDI-TOF MS) Based Methods

Matrix-assisted laser desorption ionization time-of-flight mass spectrometry (MALDI-TOF MS) is a new type of soft ionization biological mass spectrometer developed in recent years. MALDI-TOF MS has revolutionized clinical microbiology laboratories for accurate microorganism identification [48,49,50,51,52]. Because of its rapidity, accuracy, and simplicity, the application range of MALDI-TOF MS is increasing and also shows promising results in detecting antimicrobial resistance. MALDI-TOF MS-based AST approaches comprise methods that detect particular resistance mechanisms or methods for universal AST.

The modification of antibiotic structures induced by bacterial enzymes can be measured by MALDI-TOF MS and used to detect antibiotic resistance. Especially, the detection of β-lactamases [53,54] or the AAC(6’)-lb-cr enzyme [55,56] has been demonstrated in many studies [48]. However, detection of only particular resistance mechanisms is the main inherent limitation of MALDI-TOF MS-based AST methods.

In addition to ATP and nucleic acid, the expression of protein also reflects changes in cell metabolism. MALDI-TOF MS can analyze the whole cell proteome. Therefore, antimicrobial resistance can be judged by the detection of microbial proteome changes after drug treatment. This approach eliminates the subjective visual endpoint and can provide the result within a few hours [57]. Furthermore, this method is applicable to various resistance mechanisms and can be applied to different microbial species and antibiotics [58].

Lange et al. developed a rapid and phenotypic AST approach, the MALDI Biotyper antibiotic susceptibility test rapid assay (MBT-ASTRA), by quantifying the amounts of bacterial peptides using MALDI-TOF MS [59]. The quantities of these peptides, which can be evaluated by the peak intensities of mass spectra, correlate to the number of microorganisms and, therefore, to the growth of a microorganism. Clinical isolates of *Klebsiella pneumoniae* and *Klebsiella oxytoca* were used for the evaluation against meropenem. After 1 h of incubation at a meropenem concentration of 8 μg mL^-1^, the resistant strain has mass peaks that were equivalent to that of the sample incubated without meropenem. In contrast, no visible mass peaks were observed for the sensitive strain. Therefore, the resistant and sensitive strains could be distinguished. This method achieved a sensitivity of 97.3%, and a specificity of 93.5% for a total of 108 *K. pneumoniae* isolates tested against meropenem.

Toward clinical applications, this method was also evaluated for bacteria isolated from positive blood cultures in the previously mentioned and subsequent studies [60]. In a work reported by Jung et al., bacterial cells extracted from positive blood cultures were evaluated with the MBT-ASTRA approach [60]. A total of 30 blood cultures spiked with *Enterobacteriaceae* and 90 patient-derived blood cultures of Gram-negative bacteria were tested against 4 non-β-lactam antibiotics. Bacteria were classified as susceptible or non-susceptible by a relative growth value calculated as the ratio of mass spectra with and without antibiotic. The assay correctly classified the susceptibility of all bacteria tested for gentamicin and cefotaxime, with 5 mismatches for piperacillin-tazobactam. Identification of bacteria and analysis of susceptibility are possible within 4 h with this method.

**Figure 2:**
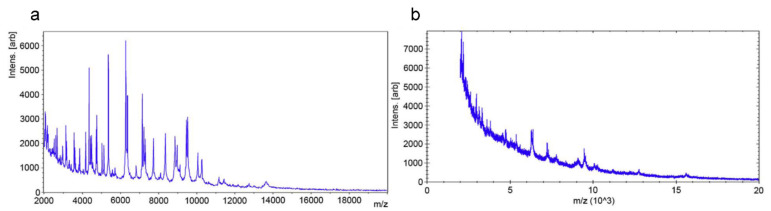
Differentiation between resistant and susceptible isolates by matrix-assisted laser desorption ionization time-of-flight mass spectrometry (MALDI-TOF MS) spectra after antibiotic incubation. (**a**) Meropenem-resistant *Klebsiella pneumoniae* isolate. (**b**) Meropenem-susceptible isolate. Reprinted from reference [61] with permission.

Idelevich et al. reported a universal phenotypic method based on MALDI-TOF MS, designated as a direct-on-target microdroplet growth assay [61]. In this method, bacterial isolates were incubated in nutrient broth with and without antibiotics directly on a disposable MALDI-TOF MS target (Figure 2). All isolates (24 *K. pneumoniae* and 24 *P. aeruginosa* isolates) tested were correctly categorized as susceptible or non-susceptible after 18 h of incubation. High accuracy could also be achieved for *K. pneumoniae* after a 4-h incubation and *P. aeruginosa* after 5-h incubation. This method is easy to perform and has the potential for high-throughput testing. However, the overall time needed to obtain the results is too long to be considered a rapid technique for clinical application.

In addition to AST using MALDI-TOF MS alone, mass spectroscopy was also combined with stable-isotope labeling to develop a universal method to determine the susceptibility of bacteria [62,63,64]. These approaches are based on bacterial growth in stable isotopes such as ^13^C and ^15^N containing media that also contain antibiotics. Characteristic mass shifts resulting from the isotopic labels being incorporated into the biomolecules were measured and used to infer antibiotic susceptibility.

Demirev et al. described a rapid method that obtains AST results in 6 h using MALDI-TOF MS and ^13^C-labeling [62]. In this study, bacteria were grown in ^13^C-labeled media with antibiotics. A mass spectrum obtained from these bacteria was compared with a mass spectrum of bacteria incubated in non-labeled media without antibiotics. The incorporation of ^13^C-labeled compounds from the media into bacterial macromolecules, for example, protein, leads to a mass shift in the spectrum. Antibiotic susceptibility was determined by the characteristic mass shifts of one or more biomarker peaks. These mass shifts were observed if the bacteria were growing in the presence of antibiotics and therefore indicated that the bacteria were resistant. Although the performance of this method is demonstrated in *E. coli* only, this method has the potential to be applied to different bacterial species since the incorporation of ^13^C-labeled compounds is a universal behavior of most bacteria.

In contrast to the previous studies that utilize completely ^13^C-labeled culture media, Sparbier et al. developed a different approach based on the incorporation of single isotopically labeled amino acids (^13^C_6_-^15^N_2_ labeled lysine) [63]. The use of only one amino acid as a labeled media compound makes the assay much more cost-effective and the evaluation less complicated. Methicillin-susceptible *S. aureus* (MSSA) and methicillin-resistant *S. aureus* (MRSA) strains were used as a model system and tested against oxacillin and cefoxitin in this study. After a 3-h incubation in normal, isotope-labeled, and isotope-labeled plus antibiotic media, mass spectra were acquired and compared. As expected, mass shifts of peaks in the spectral profile were observed in MRSA only in the presence of antibiotics. In further studies, this approach was extended to Gram-negative bacteria, *P. aeruginosa*, using antibiotics with distinct modes of action, meropenem, tobramycin, and ciprofloxacin [64]. The method works for the bacteria and antibiotics tested. However, a slightly delayed effect was observed for meropenem, as significant incorporation of the labeled amino acid was still observable in the first 90 min of incubation. To minimize this effect, a 30-min pre-cultivation of meropenem was needed before the addition of labeled and unlabeled amino acids.

The advantages of the methods described above are universality and rapidity. However, most of the AST results obtained in these works are the classification of susceptible or non-susceptible, MIC values or the intermediate criterion were not determined. Whether these methods can obtain the MIC and, however accurate the MIC results compared to the gold standard methods still need to be validated for future clinical applications. Another advantage of MALDI-TOF MS-based AST methods is the potential of integration of bacterial identification and AST in one instrument. Although the cost of a mass spectrometer is relatively high, the cost of a single AST with MALDI-TOF MS could be low, especially considering that MALDI-TOF MS has been routinely used in many clinics for identification. Compared to digital PCR-based methods, methods based on MALDI-TOF MS do not show an advantage in the time reduction. Furthermore, more procedures are needed for biomolecule extraction, and experienced performing is required. In the future, automation of procedures, integration of identification and AST are expected to expand and refine MALDI-TOF MS applications in clinical diagnosis.

### 2.4. Raman Technology-Based Methods

Raman technologies are vibrational spectroscopic techniques that have emerged as one of the major tools in biology and medicine. They have the characteristics of label-free and non-invasiveness, providing an analysis method for the study of biological samples without the need for sample extraction. In the following sections, we review the applications of Raman technologies for rapid AST.

#### 2.4.1. Raman Spectroscopy

Raman spectroscopy is molecule-specific, providing a fingerprint of the molecule. As a non-invasive real-time analysis tool, Raman spectroscopy provides information about the content and distribution of biochemical components in tissues and cells [65]. In addition, the intensity of the signal is proportional to the concentration of the molecular components [66,67]. Therefore, the change of shape and intensity of Raman spectroscopy can be used as a reflection of the change of metabolism in biological samples.

Rapid AST by Raman spectroscopy can be divided into two main approaches: the detection of the differences in characteristic spectral changes of susceptible and resistant bacteria with or without antibiotic treatment and the quantification of carbon−deuterium (C−D) peak intensity changes after deuterium labeling of bacteria and antibiotic treatment.

Raman spectra, especially the Raman peaks in the fingerprint region, have been used as a marker for AST by quantifying the spectral change upon antibiotic treatment [68,69,70,71]. In a study reported by Kirchhof et al., spectral marker bands in the fingerprint region of Raman were used to indicate the effect of ciprofloxacin toward *E. coli* [68]. They found that ciprofloxacin, a fluoroquinolone antibiotic, induced concentration-dependent changes of Raman peaks in *E. coli* after only 90-min treatment. The band at 1458 cm^−1^ increased while the shoulder band at 1485 cm^−1^ decreased with increasing ciprofloxacin concentration. By defining an intensity ratio of the bands at 1458 cm^−1^ and 1485 cm^−1^, MIC could be determined with a threshold in less than 2 h total analysis time. For a total of 15 *E. coli* strains tested, a correct sensitivity classification was obtained for 20 out of 25 measurements. The approach could obtain the MIC values in less than 2 h without the need for pre-cultivation. 

In a subsequent work by the same group, a similar approach was used to detect vancomycin resistance in *enterococci* and showed that the susceptibility (susceptible or resistant) can be determined within 3.5 h without the need for any information on strain identity [69]. In this work, two different Raman bands, around 1250 cm^−1^ and 1485 cm^−1^ were found to have characteristic changes in as short as 30 min after vancomycin addition and used as spectral markers to differentiate resistant and susceptible *enterococci*. This method achieved a sensitivity of 87% and a specificity of 93% for *E. faecalis*, and a sensitivity of 99% and a specificity of 77% for *E. faecium*. Similarly, spectroscopic measurements can be used in the absence of antibiotics to characterize acquired antibiotic resistance and the mode of action of resistance. Germond et al. reported the utility of Raman spectroscopy in detecting antibiotic resistance in *E. coli* [72]. In this work, Raman spectral peak intensities were found to correlate with the expression of some well-known antibiotic resistance genes. With Raman spectroscopy, the type of antibiotic resistance and mode of action could be identified in 11 strains in a repeatable manner. However, like genotypic AST, this method relies on the detection of known resistance mechanisms and therefore is not a universal method.

**Figure 3:**
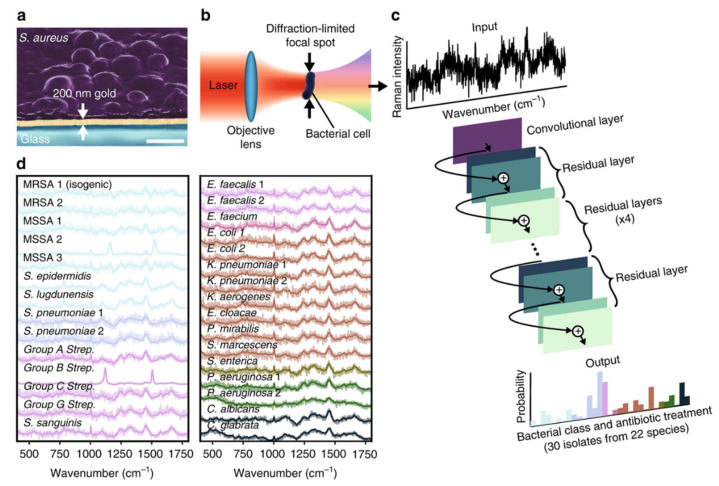
Rapid identification of bacteria and antibiotic susceptibility by Raman spectroscopy and deep learning. (**a**) Bacterial cells deposited onto gold-coated silica substrates. The scale bar is 1 μm. (**b**) Conceptual measurement schematic: by focusing the excitation laser source to diffraction-limited spot size, Raman signal from single cells can be acquired. (**c**) Classification of bacteria from low-signal Raman spectra using a one-dimensional residual network with 25 total convolutional layers. (**d**) Averages of 2000 Raman spectra from 30 isolates are shown in bold and overlaid on representative examples of noisy single spectra for each isolate. Spectra are color-grouped according to empiric antibiotic treatment. Reprinted from reference [74] with permission.

Raman spectroscopy-based AST methods described above are based on detecting subtle spectral differences phenotypically with antibiotic treatment or spectrally without antibiotic treatment. However, these spectral differences are easily masked by background noise due to the weak process of spontaneous Raman [73]. Therefore, a long integration time is typically needed to achieve high accuracy, resulting in a low-throughput single-cell measurement [74]. To address this challenge, the combination of Raman spectroscopy and deep learning provides a new solution for rapid AST. Ho et al. trained a convolutional neural network to identify bacteria and their antibiotic resistance by Raman spectra [74]. This study uses measurement times of 1 s, corresponding to signal to noise ratios (SNRs) that are an order of magnitude lower than typical reported bacterial spectra while still achieving comparable or improved identification accuracy (Figure 3). This model achieved recognition accuracy of 89.1% to distinguish methicillin-resistant and -susceptible isolates of *S. aureus* (MRSA and MSSA) in a total of 200 measurements. The advantage of this method is that it is culture-free, and thus the total assay time is constrained by sample preparation, Raman measurement, and data analysis only. Moreover, with the single-cell measurement capability, this approach has the potential to be readily extended for diagnostics on patient samples such as blood and urine.

The AST approaches based on detecting characteristic Raman spectral changes, either the specific Raman peak changes after antibiotic treatment or the differentiation of susceptible or resistant bacteria by Raman spectra, need to be evaluated case by case for different bacteria species or antibiotics and therefore are not universal methods. Several studies that combined Raman spectroscopy with stable isotope labeling to determine antimicrobial susceptibility by detecting bacterial metabolism provide an alternative solution for universal AST. Tao et al. employed Raman spectroscopy to probe bacterial response to different drugs by assessing the metabolic activity of heavy water (D_2_O)-labeled bacterial cells [75]. The mechanism is that in the presence of D_2_O, D^+^ from D_2_O can be incorporated to form C−D bonds in intracellular macromolecules in active bacterial cells. The rate of C−D bond formation can be detected by Raman spectroscopy using Raman shift at the C−D band from 2040 to 2300 cm^−1^. As the intake of H_2_O or D_2_O is a basic property of active cells, D_2_O-Raman can serve as a universal method to detect and measure cells’ metabolic activity [76,77,78]. With the D_2_O-Raman method, fluoride-sensitive and fluoride-resistant *S. mutans* strains could be discriminated in as early as 0.5 h. In addition, the idea of “minimum inhibitory concentration based on metabolic activity (MIC-MA)” was proposed and determined for *S. mutans* in two antiseptics (sodium fluoride and chlorhexidine) and one antibiotic (ampicillin) to quantify antibacterial efficacy via metabolism inhibition. However, the MIC-MA values obtained in this study tend to be higher than conventional MIC values.

Toward clinical application, Yang et al. coupled Raman spectroscopy with D_2_O-labeling and developed a metabolic activity-based rapid AST for UTI samples [79]. In this approach, clinical urine samples after a simple filtration were first incubated in media containing D_2_O and antibiotics. The antibiotic concentration of 10 × CLSI MIC breakpoint was used for the treatment. Then, a simple susceptibility/resistance (S/R) cutoff value based on the C-D ratios was established for the S/R readout. This method reduced the total test time from receiving urine samples to S/R readout to only 2.5 h.

Single-cell Raman spectroscopy has shown to be a promising tool for rapid AST. The advantages of AST with Raman spectroscopy are its rapidity, universality of the D_2_O-Raman method, and single bacterial cell measurement capability directly from clinical samples. Moreover, Raman spectroscopy is already commercially available. On the other hand, the D_2_O-Raman method can be time-consuming and of low-throughput in the measurement step. To address these challenges, the integration of deep learning into Raman spectroscopy could significantly reduce the measurement time. With this potential, the Raman spectroscopic assays could become an integral part of a novel diagnostic tool that provides MIC results within hours in the clinic.

#### 2.4.2. Stimulated Raman Scattering (SRS) Imaging

Spontaneous Raman scattering (SRS) is usually a very weak process; about 1 out of 10^8^ photons undergoes Raman scattering spontaneously [73]. This inherent weakness limits the strength of the Raman signal [80]. One way to enhance the signal of Raman scattering is the nonlinear optical processes, coherent Raman scattering. The major approaches are coherent anti-Stokes Raman scattering (CARS) and SRS. Compared to CARS, SRS is usually preferred since it does not suffer from the nonresonant background that could distort vibrational peaks.

The development of SRS microscopy has significant advances in its applications in biology and medicine. SRS has been used for metabolic imaging in cells [81,82,83], tissues [84], and model organisms [85]. With the advantages of high-speed imaging and sub-micron resolution, SRS is also suitable for bacteria study at the single bacterial cell level.

Our group first introduced SRS for rapid phenotypic AST by imaging deuterated glucose metabolism in bacteria [86]. Glucose is the preferred carbon source for most bacteria [87]; therefore, detecting glucose metabolism could provide a universal method to study the metabolic activity of bacteria. Vancomycin-susceptible *enterococci* (VSE) and vancomycin-resistant *enterococci* (VRE) were first used as models for the development of the AST method in this study. We showed that SRS could detect and quantitate deuterated glucose metabolism in a single bacterium. After incubation with a medium containing deuterated glucose and vancomycin, while the C−D intensity of VSE was reduced to about 1/2 of the average intensity in the control group, the C−D intensity of VRE did not show any significant change compared to the control. The C−D intensity change in VSE was observable within 0.5 h; therefore, the susceptibility could be determined rapidly within one cell cycle. More importantly, the MIC of bacteria was also correctly determined with this metabolic imaging method within 0.5 h. In addition, we further demonstrated that this method could accurately determine antimicrobial susceptibility in different bacterial species, including *E. coli*, *K. pneumonia*, and *S. aureus*, as well as antibiotics with different antimicrobial mechanisms.

**Figure 4:**
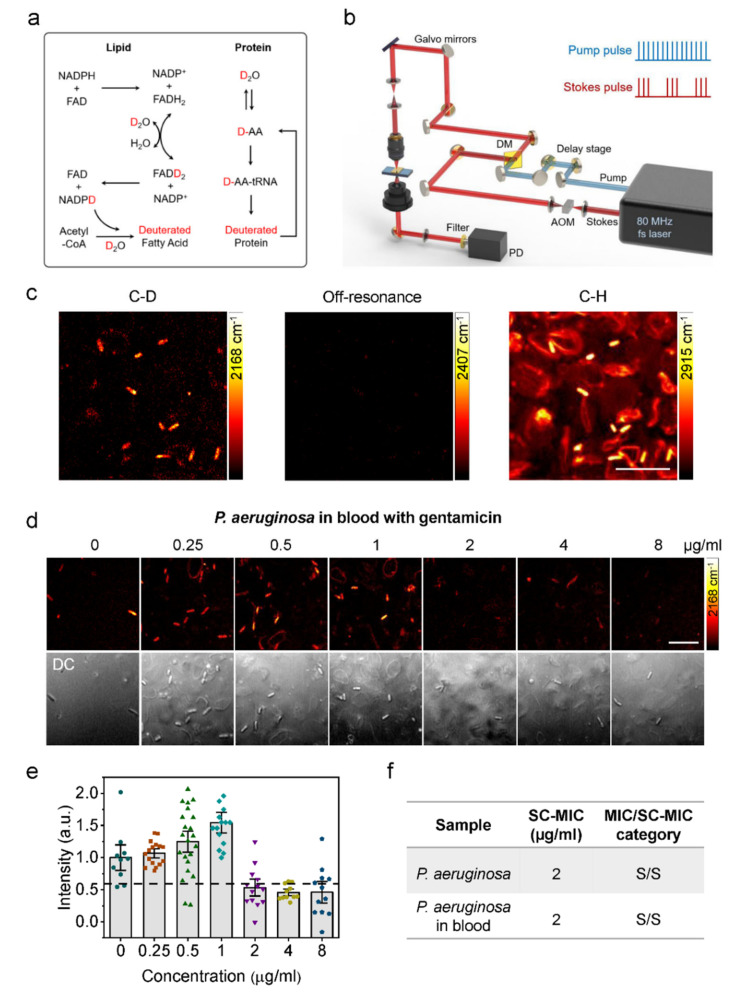
Rapid AST by SRS imaging of D_2_O metabolic incorporation. (**a**) Scheme for D_2_O labeling of lipid and protein. (**b**) SRS setup. AOM: acousto-optic modulation. DM: dichroic mirror. PD: photodiode. (**c**) SRS images at C–D, off-resonance, and C–H of bacteria in blood after 1-h culture in D_2_O containing medium. Scale bar: 10 μm. (**d**) SRS and corresponding transmission images of *P. aeruginosa* in blood after 1-h culture in D_2_O containing medium with the addition of serially diluted gentamicin. Scale bar: 10 μm. (**e**) Statistical analysis of C–D intensity in bacteria in (**d**). (**f**) Comparison of SC-MIC and susceptibility category for *P. aeruginosa* isolates and *P. aeruginosa* in blood. S: sensitive. Reprinted with permission from reference [88].

In subsequent work, we used D_2_O to incubate bacteria and demonstrated a rapid phenotypic AST method in clinic-relevant environments with SRS [88]. The C−D vibrational band, which was generated in biomolecules after D_2_O incorporation, could be selectively detected with SRS (Figure 4). We found that D_2_O-SRS can detect bacteria’s metabolic activity and their metabolic response to antibiotic treatment in as short as 10 min of incubation. Furthermore, we showed that single-cell metabolism inactivation concentration (SC-MIC), a parameter comparable to the conventional broth microdilution MIC, can also be obtained within 2.5 h with our method. Our method achieved a categorical agreement of 94.6% and an essential agreement of 86.5% in a total of 37 sets of bacterial isolate samples, which include 8 major bacterial species and 14 different antibiotics often encountered in the clinic.

Toward clinical translations, AST with D_2_O-SRS was further demonstrated for bacteria in urine and blood samples. Urine samples spiked with *E. coli* and blood samples spiked with *P. aeruginosa* were used for the proof-of-concept demonstration. By tuning SRS imaging to the C−D band, only the bacterial cells show strong C−D signals in urine and blood samples, enabling the detection and quantification of SC-MIC. With D_2_O-SRS, the total assay time of AST for bacteria in urine and blood was shortened to less than 3.5 h.

In addition to rapidity, the main advantages of SRS-based AST are universality and direct measurement for clinical samples. Compared to Raman spectroscopy-based AST methods, SRS imaging could simultaneously detect all the bacterial cells in a field of view and therefore has the potential for high-throughput measurements. On the other hand, the instrument of SRS is relatively expensive and bulky. In the future, less expensive and less bulky fiber laser-based CARS can be tested as an alternative approach for rapid AST. Furthermore, automated sample preparation and data acquisition in a multi-well chamber need to be developed to translate this approach into clinical applications.

## 3. Conclusions

The emergence of antimicrobial resistance has become a growing threat to global health. For infection diseases like septic shock, early treatment has relevance to the decrease of the death rate. Therefore, antimicrobial susceptibility needs to be determined rapidly at the point of care. To achieve this, an ideal AST would be a phenotypic method that is generalizable to different pathogens or antibiotics and provides sample-to-answer AST results in less than 30 min during a single patient visit [31]. In addition, a point of care AST method needs to work directly from clinical samples such as urine or blood to achieve this speed.

In this review, we summarized recent rapid AST methods based on the profiling of bacterial metabolism. Table 1 shows the comparison of these methods. These metabolism-based AST methods take a shorter turnaround time than the culture-based methods and could enable early clinical decisions in treating microbial infections. These methods are based on detecting ATP level, the copy number of nucleic acids, or macromolecule synthesis in bacteria. Compared to the multigeneration growth of bacteria in conventional culture-based methods, methods based on probing bacterial metabolic response to antibiotic treatment can achieve AST results faster. Many of these methods reduce the time to obtain an AST result to a few hours or even within 30 min, and thus have the potential for sample-to-answer AST results within the same working shift (8 h) or at the point of care. On the other hand, standardized protocols, integration of devices, and automated techniques need to be developed to accelerate routine diagnosis in clinical applications of these rapid AST methods.

## Figures and Tables

**Table 1:** Comparison of different existing rapid antimicrobial susceptibility testing (AST) methods based on metabolic profiling.

AST Technology	Mechanism	Time of Sample Preparation	Time to Result	Direct on Clinical Samples	Provide MIC	References	Cost for Device	Cost for a Single Test
ATP bioluminescence	ATP concentration		2 h from urine	Yes/urine	No	[27]	Low	Low
		2 h for preculture	20 min–1 h from cultured bacteria	No	No	[29]		
		15 min for centrifugation	At least 6 h from blood	Yes/ blood	Yes	[30]		
Digital PCR	Copy number of DNA		30 min from urine	Yes/urine	No	[31]	High	High
		14–18 h for preculture	2 h from cultures in log phase	No	No	[45]		
			4 h from isolates or urine	Yes/urine	No	[89]		
MALDI-TOF MS	Change of mass spectrum		2–3 h from cultured bacteria or blood	Yes/blood	No	[59]	High	Low
			4 h from blood	Yes/blood	No	[60]		
			4–18 h from cultured bacteria	No	No	[61]		
		1 h for subculture	4 h from blood	Yes/blood	No	[90]		
		20 min for centrifugation	3.5 h from blood	Yes/blood	No	[91]		
Raman spectroscopy	Change of Raman spectrum	2 h for preculture	4 h from isolates	No	Yes	[70]	High	Low
		Overnight-culture in D_2_O	At least 40 min from overnight cultures	No	Yes	[75]		
		15 min for filtration	2.5 h from urine	Yes/urine	No	[79]		
SRS	Quantify deuterium incorporation	2 h for preculture	At least 0.5 h from cultures in log phase	No	Yes	[86]	High	Low
		15 min for centrifugation and filtration	2.5 h from isolates, urine, or blood	Yes/urine and blood	Yes	[88]		

## Data Availability

No new data were created or analyzed in this study. Data sharing is not applicable to this article.
